# Tumour-associated macrophages in diffuse large B-cell lymphoma: the prognostic and therapeutic impact in a South African centre with high HIV seroprevalence

**DOI:** 10.1007/s12026-024-09537-x

**Published:** 2024-09-11

**Authors:** Jenifer Vaughan, Tracey Wiggill, Zainab Mia, Moosa Patel

**Affiliations:** 1 Department of Molecular Medicine and Haematology, Faculty of Health Sciences, University of the Witwatersrand, Johannesburg, South Africa; 2 National Health Laboratory Services, Johannesburg, South Africa; 3 Immunology Unit, Division of Medical Microbiology and Immunology, Faculty of Medicine and Health Sciences, Stellenbosch University, Cape Town, South Africa; 4 Department of Anatomical Pathology, Faculty of Health Sciences, University of the Witwatersrand, Johannesburg, South Africa; 5 Clinical Haematology Unit, Department of Medicine, Chris Hani Baragwanath Academic Hospital and Faculty of Health Sciences, University of the Witwatersrand, Johannesburg, South Africa

**Keywords:** HIV-associated lymphoma, Tumour macrophages, Tumour microenvironment, Diffuse large B-cell lymphoma, HIV-DLBCL, Rituximab

## Abstract

Diffuse large B-cell lymphoma (DLBCL) is a common malignancy among people living with HIV. Macrophage enrichment of the tumour microenvironment (TME) is a prognostic factor in DLBCL among immunocompetent people, with some studies reporting that macrophage enrichment predicts a superior response to rituximab therapy. The macrophage phenotype is also important, with reportedly poorer outcomes with enrichment of anti-inflammatory (M2) macrophages. To date, the relationship between the type/number of tumour macrophages and outcomes in HIV-associated DLBCL (HIV-DLBCL) has been poorly explored. In this study, we assessed tumour macrophage numbers in a South African cohort of patients with DLBCL and a high HIV-seropositivity rate. Immunohistochemistry for CD68 and CD163 was performed on the diagnostic biopsies of 79 patients with DLBCL. Relevant information was documented from the clinical records, including disease stage, international-prognostic index score, HIV-related parameters, C-reactive protein, ferritin levels and immune cell numbers (monocytes, lymphocytes and neutrophils). Survival analysis was performed using Kaplan–Meier survival estimates, and the correlation between tumour macrophage numbers and a variety of immunological parameters was assessed using Spearman’s rho. Of the 79 patients included, 87.2% were living with HIV, and rituximab therapy was used in 46.9%. Tumour macrophage numbers were not related to HIV status, but low pro-inflammatory (M1) macrophage numbers (CD68 + CD163 −) were significantly associated with poorer outcomes (HR 2.02, *p* = 0.03). M2 macrophage (CD68 + CD163 +) enrichment was not predictive of survival but was associated with improved response to rituximab therapy (HR 0.19; *p* = 0.002). Macrophage numbers were marginally correlated with ferritin levels, which showed modest performance as a peripheral blood biomarker of the TME macrophage status (AUC 0.6 at a level of 374 µg/L), and high ferritin levels were associated with a superior response to rituximab-therapy (HR 0.28, *p* = 0.034). Pro-inflammatory macrophages are important in tumour control in HIV-DLBCL, while M2 macrophage enrichment improves the response to rituximab therapy. Ferritin shows promise as a biomarker for identifying patients more likely to benefit from rituximab therapy.

## Introduction

Diffuse large B-cell lymphoma (DLBCL) is a lymphoid neoplasm, which is among the most common subtypes of non-Hodgkin lymphoma encountered among people living with HIV. Its outcomes depend on clinical factors (including patient age and performance status, clinical stage and serum LDH levels), tumour biology (cell of origin (COO), genetic profile, immunophenotype) and the underlying tumour microenvironment (TME). The latter has been demonstrated in both gene expression profiling [1] and immunohistochemical (IHC) studies, where tumour enrichment by macrophages has been found to be a significant prognostic factor [2–6]. Macrophages are tissue-resident phagocytes which play an integral role in the innate immune system [7]. They can be broadly subdivided into two functional subgroups: classically activated (M1) macrophages, which have a pro-inflammatory phenotype and function in anti-microbial defence, and alternatively activated (M2) macrophages, which produce immunosuppressive cytokines (such as interleukin (IL)10 and transforming growth factor β (TGF-β)), and function to dampen immune responses following a pro-inflammatory stimulus [7]. While all macrophages express the lysosomal glycoprotein CD68, the macrophage scavenger receptor CD163 is dominantly expressed by M2 macrophages, which allows for these cells to be distinguished on immunohistochemistry [8].

In cancer, M1 and M2 macrophage polarisation is directed according to the dominant cytokine profile in the TME, which in turn is determined by growth factors and chemokines made by the tumour cells as a result of intrinsic oncogenic genetic events, or as a consequence of extrinsic immune activation secondary to underlying inflammation [9]. The tumour-associated macrophages (TAMs) are originally predominantly of the M1 (pro-inflammatory) subtype but undergo phenotypic switching due to sustained cytokine exposure [10]. M2 macrophages can also arise from monocytes in peripheral circulation, a process promoted by the enzyme Indoleamine 2,3-dioxygenase (IDO) [11]. IDO catalyses the conversion of the amino acid tryptophan to kynurenine, and the resultant tryptophan depletion causes pro-inflammatory T-cell apoptosis, as well as induction of resting T-cell differentiation into regulatory T-cells (Tregs) [11]. In the context of cancer, a M2 predominant macrophage repertoire is thought to negatively impact outcomes owing to depression of the anti-tumour immune response (i.e. tumour tolerance). In line with this, several studies have shown the negative outcomes observed in TAM-enriched tumours to be a product of increased M2 macrophage numbers [2–4]. Furthermore, some studies have demonstrated a potential for the number of TAMs to affect the therapy response in DLBCL, with significantly improved outcomes in cases with TAM-enriched tumours where the anti-CD20 monoclonal antibody rituximab was included in the therapy protocol [5]. This is thought to be due to enhanced phagocytic capacity for the destruction of antibody-coated tumour cells where macrophages are abundant.

To date, the relationship between TAM numbers and outcomes in HIV-associated DLBCL (HIV-DLBCL) has been poorly explored, but protracted infection-related (extrinsic) immune activation could conceivably promote tumour-enrichment with M2 macrophages in this setting. This may in turn contribute to the less favourable outcomes sometimes observed in this entity. A single study in this regard showed no significant difference in TAM numbers according to HIV status among patients with DLBCL and no significant relationship between TAM numbers and survival, although particularly poor outcomes were noted in the small number of patients with very low TAM numbers [12]. We have previously confirmed significant negative associations between survival and elevated peripheral blood levels of IL10, TGFB and high Treg numbers in a South African cohort of patients with DLBCL and a high HIV seropositivity rate [13, 14]. In this study, we aimed to assess M2-TAM numbers and their association with survival in our setting. As a secondary endpoint, we aimed to assess a spectrum of peripheral blood biomarkers (including immune cell numbers, cytokines and other immunological proteins) as a surrogate for histological assessment of TAM numbers, since this is a labour-intensive and poorly standardised procedure.

## Methods

**Fig. 1:**
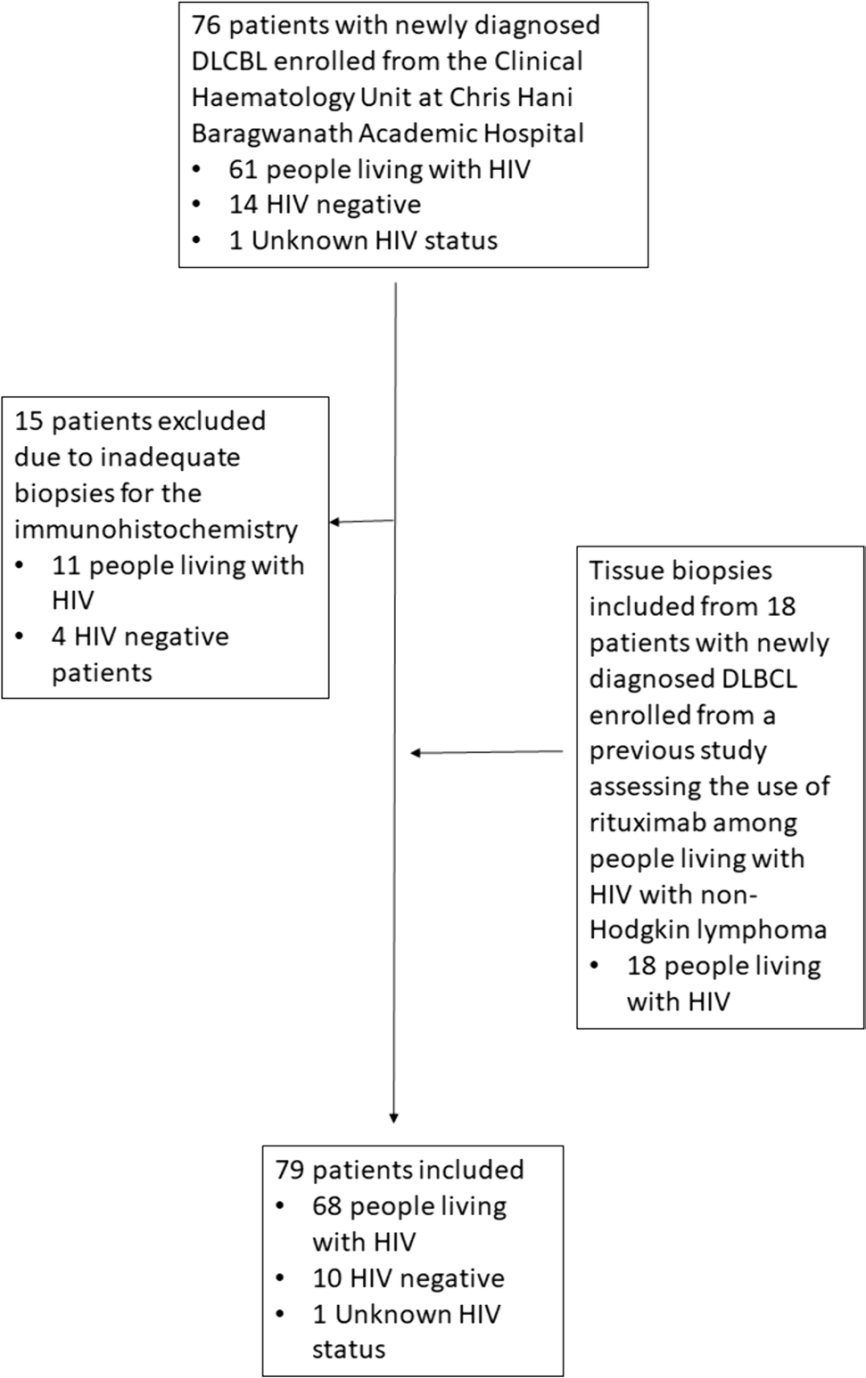
A flow diagram depicting the patients included in this study

Seventy-six adult patients with newly diagnosed DLBCL at the Chris Hani Baragwanath Academic Hospital (CHBAH) in Johannesburg were enrolled between November 2019 and April 2022 (as previously reported) [13, 14]. Of these, 15 were excluded as they had insufficient/unavailable residual diagnostic tissue for further analysis. Testing of peripheral blood IL10, IL6, TGFB and IDO activity (as evidenced by the kynurenine:tryptophan ratio) and Treg numbers was performed on selected patients (as reported previously [13, 14]). Since the number of patients treated with rituximab was small in this cohort, a further 18 samples collected from patients with HIV-DLBCL were sourced from a previous as yet unpublished study entitled “A multi-centre, prospective, randomised study of the efficacy and safety of Rituximab (R) in combination with Cyclophosphamide, Hydroxydaunorubicin/Adriamycin, Oncovin/Vincristine, Etoposide, Prednisone (i.e. R-CHOEP) in comparison to CHOEP alone, in previously untreated (newly diagnosed) adult patients with Human Immunodeficiency Virus (HIV) related lymphomas in South Africa”. Only patients with DLBCL in the earlier study were included in the current study, both those who received CHOEP and those who received R-CHOEP upfront. A flow diagram showing patient inclusion and exclusion for the current study is depicted in Fig. 1. Both of these studies, as well as the further subanalysis, were approved by the Human Research Ethics Committee of the University of the Witwatersrand (references M190709 and M1211108, respectively). Informed consent was obtained from all participants from whom peripheral blood was prospectively collected for the purposes of the study. Informed consent was not invariably obtained from patients where testing was performed on residual blood or tissue samples submitted for routine laboratory tests but was acquired if the patients had not demised or been lost to follow-up in the interim.

### Immunohistochemistry

**Fig. 2:**
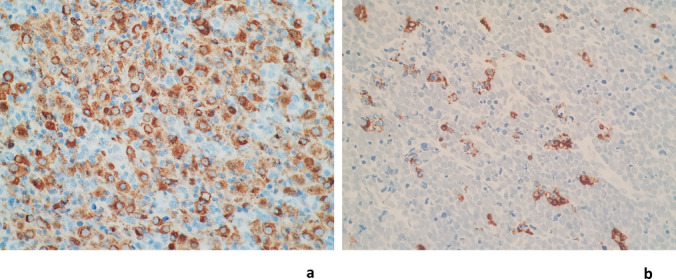
A depiction of cases with high (**a**) and low (**b**) macrophage numbers. Stained with CD68, 100 × magnification

TAM numbers were assessed on the diagnostic formalin-fixed paraffin-embedded tissue samples by immunohistochemistry for CD68 (all TAMs) (monoclonal anti-CD68 antibody; clone PG-M1, Dako Cytomation, Denmark) and CD163 (M2-TAMs) (monoclonal anti-CD163 antibody; 1:200, MRQ-26 clone, Cell Marque, Sigma-Aldrich Co, CA, USA) using similar methods to those described by Riihijarvi et al. [5]. Analysis was performed using an Olympus BX40 light microscope (Olympus Corporation, Tokyo, Japan). Briefly, three to five representative fields (depending on the tissue available) with the most abundant TAMs without necrosis or fibrosis were photographed. The number of CD68 + and CD163 + TAMs were counted manually from the captured images as the absolute number of CD68 + or CD163 + cells per field at 800 × magnification (field of view approximately 0.08 mm^2^) and then averaged. To mitigate the possibility of inter-observer variation, analysis was performed by a single pathologist (author JV). The number of M1 macrophages was calculated as the difference between the number of CD68-positive cells (all macrophages) and the number of M2 macrophages (CD68 + CD163 +). Representative images of cases with high and low TAM numbers are shown in Fig. 2. Other relevant information was documented from the clinical and laboratory records where available, including patient age and ECOG performance status, disease stage, CD4 counts and HIV viral loads (HIVVL), pertinent drug exposures (antiretroviral therapy (ART) and chemotherapy), histological details (including COO as per the Hans algorithm), full blood counts and differential white cell counts, serum LDH, C-reactive protein (CRP) and Ferritin levels, and the IPI score.

### Statistical analysis

Results are presented as medians (interquartile ranges) and proportions as appropriate. The cut-off value for high and low macrophage numbers per photographed field was determined as the level with the highest sensitivity and specificity on the receiver operating characteristics (ROC) curve analysis for predicting death. Continuous and categorical variables were compared with the Mann Whitney *U* and Fisher’s exact tests, respectively, and correlation analysis was performed using Spearman’s rho. The 12-month survival rate was calculated as the proportion of patients alive 12 months after diagnosis, and survival time was compared using Kaplan–Meier survival estimates and log-rank tests. Multivariate survival analysis of factors identified to have a significant association with survival on Kaplan–Meier analysis was performed using Cox proportional hazards analysis in order to identify factors independently associated with survival. Schoenfield analysis of proportionality was tested at https://acetabulum.dk/cgi-bin/cox, Cox proportional analysis was performed at https://statpages.info/prophaz.html, and all other analysis was performed using Prism software, version 5 (GraphPad Software, San Diego, CA, USA).

## Results

Of the 79 patients included in this study, HIV status was known in 78, of whom 68 (87.2%) were people living with HIV. ART status was documented in 64 patients, of whom 54 (84.4%) were on ART, although this had been initiated after the onset of lymphoma-related symptoms in 15 patients (27.8%). The people living with HIV were significantly younger than the HIV-negative patients, and their CD4 counts were marginally lower. No significant differences in the frequency of stage IV disease, an IPI score of 4 or more, LDH levels, COO or survival were seen according to HIV status. Survival was significantly linked to low CD4 counts and high IPI scores, but not to the presence of virological suppression. Among the 64 patients known to have received chemotherapy, only 18 (28.1%) were treated with a rituximab-containing regimen upfront, but further 12 patients (18.8%) received rituximab during second-line treatment for an incomplete therapy response. Pertinent clinical information is shown in Table 1.

**Table 1:** Pertinent clinical data

Parameter	Result (all patients)	People living with HIV	HIV-negative patients	*p*-value*
Age, median (IQR)	41 (34–52)	40.5 (34–49.3)	57.5 (34.8–64)	**0.049**
Male to female	1.05:1	1:1	1.5:1	0.74
HIV seropositive, *n*/*N* (%)	68/78^#^ (87.2)	/	/	/
CD4 (cells/µl), median (IQR)	182 (78–301) (*n* = 76)^#^	173 (73–257) (*n* = 68)	303 (146–787) (*n* = 7)	0.065
HIVVL (cps/ml), median (IQR) (*n* = 64)	151 (20–101550)	151 (20–101550)	/	/
Virological suppression^ψ^, *n*/*N* (%)	26/64 (40.6%)	26/64 (40.6%)	/	/
ART exposure, *n*/*N* (%)	54/64 (84.4%)	54/64 (84.4%)	/	/
Stage IV disease, *n*/*N* (%)	50/68 (73.5)	45/58 (77.6) §	5/10 (50.0)	0.11
IPI 4–5, *n*/*N* (%)	16/65 (24.6)	12/55 (21.8) §	4/10 (40.0)	0.25
Performance status ≥ 2, *n*/*N*(%)	22/63 (34.9)	18/54 (33.3) §	4/9 (44.4) §	0.71
Bulky disease	29/60 (48.3)	23/51 (45.1) §	6/9 (66.7) §	0.29
Extranodal disease at 2 or more sites Ϸ, *n*/*N* (%)	37/71 (52.1) #	33/60 (55.0) §	4/10 (40.0)	0.50
Cell of origin GC^α^, *n*/*N* (%)	40/58 (69)#	35/50 (70) §	5/7 (71.4) §	0.66
Ki-67 (%), median (IQR)	90 (85–95) (*n* = 73) §	90 (83.8–95) (*n* = 62) §	90 (81–99) (*n* = 10)	0.82
LDH (U/L), median (IQR)	636 (394–1286) (*n* = 75) §	636 (411–1274) (*n* = 65) §	665 (301–1437) (*n* = 10)	0.63
Β_2_microglobulin (mg/L), median (IQR)	4.7 (2.95–6.80) (*n* = 61) §#	4.8 (3.1–6.8) (*n* = 53) §	3.7 (2.7–7.6) (*n* = 8) §	0.54
Ferritin (µg/L), median (IQR)	306.5 (129.3–861.5) (*n* = 64)	303 (130–868) (*n* = 55) §	386 (91–906.5) (*n* = 9) §	0.86
Index chemotherapy protocol, *n*/*N* (%)				/
CHOP/CHOEP-based	42/64 (65.6)			
R-CHOP/R-CHOEP-based	18/64 (28.1)			
Other combination chemotherapy regimens	4/64 (6.3)			
One-year survival, *n*/*N* (%)	30/78 (38.5) §	27/68 (39.7)	3/10 (30)	0.73

*HIVVL* HIV viral load, *ART* antiretroviral therapy, *IPI* international prognostic index, *CHOP* cyclophosphamide, doxorubicin, vincristine and prednisone, *R-CHOP* rituximab plus CHOP, *GC* germinal centre subtype. *p-values derived from analysis of HIV + vs HIV − . #, one patient had an unknown HIV status; ψ, HIVVL < 100 cps/ml; §, some clinical information/laboratory results were not available in all patients; *α*, according to the Hans algorithm. The balance of the cases were of non-GC origin. Ϸ, extranodal disease includes cases with bone marrow, liver and spleen involvement

Bold entries indicate statistical significance

### Macrophage results

In this study, there was a slight predominance of M1 macrophages (CD68 + CD163 −) (median ratio M1 to M2 ratio = 1.18:1) (Table 2). The number of M2 macrophages appeared relatively higher among the people living with HIV, but this difference lacked statistical significance, possibly due to the small number of HIV-negative individuals included (Table 2). Both M1 and M2 macrophage numbers showed marginally significant correlations with the ferritin level (Table 3), and M2 macrophage numbers showed an additional significant correlation with IL10 levels and a marginally significant correlation with the tryptophan levels (Table 3). None of the macrophage numbers (global, M1 or M2) showed any significant correlation to any pro-inflammatory markers (IL6 or CRP), immune cell numbers (lymphocytes, monocytes, neutrophils and Tregs) or HIV-related parameters (CD4 count or HIVVL), and while there was a significant association between the ferritin and the haemoglobin levels, there was no significant relationship between the haemoglobin and the macrophage numbers (Table 3). On survival analysis, there were significant associations between lower global CD68 + and M1 macrophage numbers and a shorter survival time, while M2 macrophage numbers showed no significant relationship with survival (Table 4). The influence of global macrophage numbers was found to be marginally independent of the IPI and CD4 count on multivariate analysis (Table 5), while low M1 macrophage numbers were significantly associated with inferior survival independently from these variables (Table 6). M2 macrophage numbers were significantly higher among patients with low M1 macrophage numbers (51.75 versus 39.1; *p* = 0.017), forming the predominant macrophage subtype in 29/37 (78.4%) of these patients (as compared to 6/36 (14.3%) of cases with high M1 numbers). On survival analysis according to treatment, a clear benefit from rituximab use in the index chemotherapy regimen was not evident in general (Table 4), nor was there an obvious benefit from upfront rituximab therapy according to the number of macrophages. However, patients with high global macrophage (CD68 +) numbers showed improved survival if rituximab therapy was received at all (1st or 2nd line). On further analysis, this was more strongly associated with high M2 macrophage numbers, particularly in the people living with HIV (Table 4). Patients with low macrophage numbers generally showed no significant benefit from rituximab therapy, the exception being the people living with HIV with low M1 macrophage numbers (possibly due to correspondingly higher M2 macrophage numbers in these patients).

**Table 2:** Macrophage data

Parameter	Result (all patients) *n* = (79)	People living with HIV (*n* = 68)	HIV-negative patients (*n* = 10)	*p*-value*
Absolute CD68 numbers (all macrophages), median (IQR)	117.2 (82.4–161)	125.9 (83.5–162)	111.2 (73.7–138.5)	0.38
Absolute CD68 + CD163 − (M1 macrophage) numbers, median (IQR)	63.2 (31.8–98.2)	64.3 (33.2–100.2)	60.2 (31.4–90.1)	0.78
Absolute CD163 (M2 macrophage) numbers, median (IQR)	46.6 (29.4–75.6)	49.1 (33.1–75.6)	31 (18.3–70.2)	0.28
M1:to M2 ratio	1.18 (0.53–3.18)	1.16 (0.54–3.16)	1.81 (0.95–3.37)	0.47

**Table 3:** Results of Spearman correlation analysis

	M1 macrophages (r_s_ (*p*-value))	M2 macrophages (r_s_ (*p*-value))
Ferritin	− 0.21 (0.093)	0.22 (0.079)
Interleukin 6	0.123 (0.517)	0.021 (0.914)
Interleukin 10	− 0.217 (0.25)	0.37 (0.043)
Transforming growth factor β	− 0.084 (0.472)	− 0.136 (0.575)
C-reactive protein	− 0.187 (0.13)	0.043 (0.728)
Lactate dehydrogenase levels	− 0.021 (0.861)	− 0.097 (0.409)
Haemoglobin	0.05 (0.664)	− 0.068 (0.550)
Lymphocyte count	− 0.086 (0.449)	− 0.055 (0.632)
Monocyte count	− 0.128 (0.259)	0.010 (0.929)
Neutrophil count	0.020 (0.859)	− 0.054 (0.639)
Regulatory T-cell count	0.225 (0.301)	− 0.107 (0.628)
Kynurenine levels	0.008 (0.955)	− 0.006 (0.968)
Tryptophan levels	0.147 (0.324)	− 0.274 (0.051)
Kynurenine to tryptophan ratio	− 0.131 (0.36)	0.176 (0.216)
CD4	− 0.144 (0.215)	− 0.003 (0.977)
HIVVL	0.002 (0.988)	0.022 (0.864)

**Table 4:** Survival analysis

	Median survival (months)	Median survival in people living with HIV (months)
IPI		
≥ 4	2.75	1.75
< 4	44	44
Hazard ratio (CI)	4.60 (1.80–11.70)	5.18 (1.73–15.51)
*p*-value	**0.0014** (*N* = 65)	**0.0033** (*N* = 55)
CD4		
< 150 cells/µl	3	2.75
≥ 150 cells/µl	44	44
Hazard ratio (CI)	2.78 (1.49–5.19)	2.70 (1.38–5.27)
*p*-value	**0.0013** (*N* = 75)	**0.0037** (*N* = 68)
Virological suppression		
No	7	7
Yes	Not reached	Not reached
Hazard ratio (CI)	1.9 (0.95–3.80)	1.9 (0.95–3.80)
*p*-value	0.26 (*N* = 64)	0.26 (*N* = 64)
Rituximab exposure 1st line		
No	14	44
Yes	Not reached	Not reached
Hazard ratio (CI)	1.46 (0.66–3.2)	1.60 (0.62–4.07)
*p*-value	0.35 (*N* = 64)	0.33 (*N* = 54)
Rituximab exposure 1st or 2nd line		
No	5	10
Yes	Not reached	Not reached
Hazard ratio (CI)	2.34 (1.12–4.88)	2.51 (1.14–5.54)
*p*-value	0.024 (*n* = 64)	0.023 (*n* = 54)
Absolute CD68 + numbers* (all macrophages)		
< 89.4	3.75	3.25
≥ 89.4	44	44
Hazard ratio (CI)	2.68 (1.32–5.44)	2.94 (1.37–6.31)
*p*-value	**0.0065** (*N* = 78)	**0.0058** (*N* = 68)
Absolute CD68 + , CD163 − (M1 macrophage) numbers*		
< 58.48	4	4
≥ 58.48	44	44
Hazard ratio (CI)	2.02 (1.09–3.73)	1.82 (0.95–3.51)
*p*-value	**0.03** (*N* = 78)	0.07 (*N* = 68)
Absolute CD163 + (M2 macrophage) numbers*		
< 35.8	5	5
≥ 35.8	10	14
Hazard ratio (CI)	1.21 (0.65–2.25)	1.35 (0.68–2.67)
*p*-value	0.55 (*N* = 78)	0.39 (*N* = 68)
Low CD68 + numbers (< 89.4)*		
No rituximab exposure (1st or 2nd line)	3.25	3.25
Rituximab exposure (1st or 2nd line)	13.0	13.0
Hazard ratio (CI)	2.28 (0.79–6.59)	2.39 (0.62–9.15)
*p*-value	0.13 (*N* = 16)	0.20 (*N* = 13)
High CD68 + numbers (> 89.4)*		
No rituximab exposure (1st or 2nd line)	10	10
Rituximab exposure (1st or 2nd line)	Not reached	Not reached
Hazard ratio (CI)	2.56 (1.02–6.42)	2.59 (0.97–6.95)
*p*-value	**0.045** (*N* = 48)	0.059 (*N* = 41)
High M1 numbers (> 58.48)*		
No rituximab exposure (1st or 2nd line)	10	14
Rituximab exposure (1st or 2nd line)	Not reached	Not reached
Hazard ratio (CI)	2.89 (1.01–8.25)	2.14 (0.73–6.30)
*p*-value	**0.048** (*N* = 37)	0.17 (*N* = 32)
Low M1 numbers (< 58.48)*		
No rituximab exposure (1st or 2nd line)	3.75	3.75
Rituximab exposure (1st or 2nd line)	13	Not reached
Hazard ratio (CI)	3.36 (0.79–6.59)	3.36 (1.02–11.02)
*p*-value	0.13 (*N* = 27)	**0.046** (*N* = 22)
High M2 numbers (> 35.8)*		
No rituximab exposure (1st or 2nd line)	4.88	4.88
Rituximab exposure (1st or 2nd line)	Not reached	Not reached
Hazard ratio (CI)	5.02 (1.80–14.0)	6.58 (2.23–19.44)
*p*-value	**0.002** (*N* = 39)	**0.00077** (*N* = 35)
Low M2 numbers (< 35.8)*		
No rituximab exposure (1st or 2nd line)	10	Not reached
Rituximab exposure (1st or 2nd line)	6.25	5.63
Hazard ratio (CI)	1.05 (0.35–3.08)	0.68 (0.20–2.27)
*p*-value	0.94 (*N* = 25)	0.53 (*N* = 19)
High ferritin (> 374 µg/L)		
No rituximab exposure (1st or 2nd line)	2.75	2.75
Rituximab exposure (1st or 2nd line)	9	Not reached
Hazard ratio (CI)	3.51 (1.10–11.20)	4.04 (1.01–16.14)
*p*-value	**0.034** (*N* = 20)	**0.048** (*N* = 15)
Low ferritin (< 374 µg/L)		
No rituximab exposure (1st or 2nd line)	Not reached	Not reached
Rituximab exposure (1st or 2nd line)	Not reached	Not reached
Hazard ratio (CI)	2.15 (0.58–8.04)	1.71 (0.48–6.14)
*p*-value	0.25 (*N* = 33)	0.41 (*N* = 29)

*IPI* international prognostic index. *All macrophage numbers quoted are per photographed field. Note: survival data was not available in one patient

Bold entries indicate statistical significance

**Table 5:** Cox proportional hazards regression analysis for independent associations between the survival time and the CD68 numbers, the CD4 count and the IPI

	Coefficient	95% confidence interval	*p*-value	Hazard ratio	95% confidence interval
CD68 < 89.4	0.68	− 0.07 to 1.43	0.076	1.97	0.93 to 4.17
IPI ≥ 4	0.86	0.93 to 1.64	**0.028**	2.37	1.10 to 5.13
CD4 < 150 cells/µl	0.58	− 0.16 to 1.31	0.125	1.78	0.85 to 3.72

Bold entries indicate statistical significance

**Table 6:** Cox proportional hazards regression analysis for independent associations between the survival time and the M1 macrophage (CD68 + , CD163 −) numbers, the CD4 count and the IPI

	Coefficient	95% confidence interval	*p*-value	Hazard ratio	95% confidence interval
CD68 + CD163 − < 58.48	0.96	0.25 to 1.67	**0.008**	2.62	1.29 to 5.33
IPI ≥ 4	0.93	0.14 to 1.72	**0.02**	2.53	1.14 to 5.61
CD4 < 150 cells/µl	0.75	0.02 to 1.47	**0.04**	2.11	1.02 to 4.36

Bold entries indicate statistical significance

### Ferritin as a biomarker for tumour macrophage numbers

As the only parameter detected to have any correlation with M1 macrophage numbers, we assessed the performance of serum ferritin as a peripheral blood biomarker for tumour enrichment with M1 macrophages by means of ROC curve analysis. This demonstrated serum ferritin to have modest predictive capacity for low M1 macrophage numbers (AUC 0.60) with a sensitivity of 55.2% and specificity of 62.8% at a cut-off level of > 374 µg/L. As an easily measured and inexpensive parameter, we assessed serum Ferritin at a level over 374 µg/L as a prognostic marker complementary to the IPI and CD4 count. Among patients with lower risk features (IPI < 4 and a CD4 count > 150 cells/µl), those with a ferritin level > 374 µg/L had significantly poorer median survival times (9 months) as compared to those with lower ferritin levels (median survival time not reached, HR 9.32 (1.14–76.18), *p* = 0.037). In addition, since there was a positive correlation between the ferritin levels and M2 macrophage numbers, we assessed the value of rituximab therapy in relation to ferritin levels. This revealed significantly longer survival among patients with high ferritin levels treated with rituximab (either as a first-line or second-line agent), while survival was not significantly different among patients treated with and without rituximab if their ferritin level was < 374 µg/L (Table 4).

## Discussion

In this study on a cohort of South African patients with DLBCL and a high HIV seropositivity rate, M1 macrophages predominated by a small margin within the TME. While M2 macrophage numbers appeared relatively higher among the people living with HIV, this finding lacked statistical significance (possibly owing to the small number of HIV-negative patients enrolled). M1 macrophage numbers did not differ according to HIV status but were significantly and independently associated with survival. These findings differ somewhat from those of Liapis et al., who also reported no significant association between macrophage numbers and HIV status, but found that macrophage numbers were not associated with survival in HIV-DLBCL [12]. In contrast to previous reports [2–4], survival in relation to macrophage numbers was strongly associated with low M1 and not high M2 macrophage numbers. This suggests a dominant role of the M1 pro-inflammatory anti-cancer immune response in HIV-DLBCL and would seem to downplay the negative impact of the M2-mediated anti-inflammatory effect provided sufficient numbers of M1 macrophage are present.

Surprisingly, there was no relationship between either pro-inflammatory markers (such as CRP and IL6) or HIV-related parameters and macrophage numbers of any subtype, although ferritin levels were marginally associated with both M1 (inversely so) and M2 numbers. The lower ferritin levels in cases with higher M1 macrophage numbers are perplexing, as this goes contrary to the prevailing dogma that ferritin levels are higher in M1 macrophages, which sequester iron to make it less bioavailable to pathogens and tumour cells [15]. In contrast, M2 macrophages typically release iron to the surrounding stroma/circulation through ferroportin-mediated iron egress and store less iron as ferritin [15]. The higher ferritin levels in cases with lower M1 and higher M2 macrophage numbers in this study are thus unexpected. However, ferritin expression by M2 macrophages has been described previously in the setting of breast cancer, where it was a negative prognostic marker in lymph node–negative disease [16]. Furthermore, there is some evidence that ferritin may drive M2 macrophage polarisation in the context of wound healing [17], possibly as part of the homeostatic negative feedback response to a pro-inflammatory stimulus. Escalating ferritin levels could thus result in diminished M1 macrophage numbers as they are switched to M2 macrophages. Ferritin is known to have anti-oxidant activity, potentially protecting cancer cells from reactive oxygen species and chemotherapy-related cytotoxicity [18]. Since ferritin [19] and cytokines (including IL10 [20–22]) are known to be produced by some tumour cells, the high ferritin and M2 macrophage numbers here could also reflect paracrine manipulation of the immune response by the tumour cells owing to the tumour biology.

Since IL10 is well known to both drive M2 macrophage polarisation and to be produced by these cells, it was not surprising that the number of M2 macrophages was significantly correlated with IL10 levels. However, while we have previously shown high IL10 levels to be significantly associated with inferior survival, M2 macrophage numbers were not. Similarly, M2 macrophage numbers were marginally correlated with ferritin levels, a factor we showed previously to also be significantly and independently associated with survival in HIV-DLBCL [14]. Seemingly, the negative impact of IL10 and ferritin is not due to M2 macrophage–mediated tumour tolerance.

Rituximab therapy is now well recognised to improve outcomes in HIV-DLBCL [23–25]. Despite this, rituximab has not generally been routinely used for first-line treatment of HIV-DLBCL in our setting owing to resource constraints and lingering concerns about the compounding immunosuppressive effect of this drug. Here, we showed improved survival among patients treated with rituximab either in their first- or second-line chemotherapy protocols, with no significant difference in survival between patients treated with or without rituximab upfront. This finding suggests that rituximab can potentially be safely reserved for patients with HIV-DLBCL without a complete response to CHOP-based therapy in the resource-constrained environment. We have also shown a significant relationship between the response to rituximab and the number of macrophages in the TME. This agrees with the findings of Riihijarvi et al., who reported that increased numbers of TAMs identified a sub-set of patients with particularly poor outcomes if treated with multi-agent chemotherapy alone, but with significantly better outcomes compared to patients with low TAM numbers when rituximab was included in the therapy protocol [5]. In addition, we found that this association related particularly to the number of M2 macrophages in the TME, suggesting that the efficacy of rituximab is particularly dependent on these cells. This hypothesis is supported by the findings of Leidi et al., who showed a 2–3 fold greater phagocytic capacity of rituximab-opsonised leukaemic targets by M2 macrophages as compared to M1 macrophages in vitro, which was further amplified by the addition of IL10 [26]. This beneficial effect could then explain the lack of the anticipated negative prognostic association with M2 macrophage enrichment.

Unfortunately, macrophage enumeration by automated cell scanners is plagued with difficulties owing to the irregular shape and inhomogeneous staining of these cells and is therefore best performed manually [27]. As such assessment of macrophage enrichment is laborious and somewhat subjective and is consequently not routinely performed. Working on the hypothesis that macrophages would prove to be prognostically important in HIV-DLBCL, we also measured a number of potential peripheral blood biomarkers of macrophage numbers (including several cytokines, monocyte fluorescence and HLA-DR expression, Treg numbers, IDO activity, immune cell numbers (including monocytes, lymphocytes and neutrophils), ferritin and CRP levels). Of these, the only marker which showed any relationship to M1 macrophage numbers was the serum ferritin level, which showed modest performance as a biomarker for low M1 macrophage number prediction (AUC 0.60, sensitivity 55.2%, specificity 62.8%) at a level of > 374 µg/L. Among patients with lower risk features (IPI < 4 and a CD4 count > 150 cells/µl), those with a ferritin level > 374 µg/L had significantly shorter median survival times. Moreover, since ferritin levels were positively correlated with M2 macrophage numbers, we assessed the value of the serum ferritin in predicting the response to rituximab therapy and demonstrated a significant benefit from this agent among patients with high, but not low, serum ferritin levels. Rituximab therapy could potentially be reserved for patients with high ferritin levels in resource-restrained clinical settings and could improve outcomes among patients with high ferritin levels if used upfront. These findings emphasise the value of the serum ferritin level, an inexpensive and readily available blood test, to refine prognostic stratification and therapy selection in HIV-DLBCL, incorporating a biomarker of an important aspect of the TME in a logistically feasible manner. Further studies validating these findings are however required.

Also of interest is that a number of preclinical studies have evaluated strategies to promote macrophage repolarisation to a pro-inflammatory (M1) phenotype, including the use of iron supplementation, genetic reprogramming of M2 macrophages using injectable mRNAs, oxygen-producing nanoparticles, modulation of glutamine metabolism, glycolytic inhibitors and Toll-like receptor agonists (among others) [28]. Such strategies may prove useful in boosting M1 macrophage numbers in HIV-DLBCL in future.

Important limitations to this study include the small number of HIV-negative patients included as a comparator group, the incomplete data in a proportion of the patients and the somewhat subjective nature of manual macrophage enumeration.

## Conclusion

In this study, we have shown that TAM numbers do not differ significantly according to HIV status in HIV-DLBCL, but low M1 macrophage numbers are associated with poorer outcomes. M2 macrophage numbers were not predictive of survival but were associated with improved response to rituximab therapy. Both M1 and M2 macrophage numbers were correlated with ferritin levels, which showed modest performance as a peripheral blood biomarker of the macrophage status in the TME. Owing to the relationship between high ferritin levels and increased M2 macrophage numbers, ferritin levels showed promise for the ready identification of patients more likely to benefit from rituximab therapy, but this finding requires further validation.

## Data Availability

The data underpinning this research is available from the corresponding author upon reasonable request.
